# A digital health intervention: development and validation of a social media nursing program for sexual dysfunction following cervical cancer radical hysterectomy

**DOI:** 10.3389/fpubh.2025.1720263

**Published:** 2025-12-04

**Authors:** Chen Huang, Yanru Qiu, Xuan Huang, Hong Tang

**Affiliations:** 1 College of Literature Art, Shihezi University, Shihezi, Xinjiang, China; 2 Wuxi Medical College, Jiangnan University, Wuxi, Jiangsu, China; 3 Kashi University Medical College, Kashi City, Xinjiang, China; 4 Department of Laboratory Medicine, Jiangnan University Hospital, Wuxi, Jiangsu, China

**Keywords:** digital health, health communication, social media, cervical cancer, sexual dysfunction, health literacy, health empowerment, nursing intervention

## Abstract

**Objective:**

This mixed-methods study utilized an exploratory sequential design to develop and evaluate a digital health intervention delivered via social media for sexual dysfunction following cervical cancer surgery. The intervention aimed to improve sexual function and promote health empowerment—conceptualized as the knowledge, skills, and self-efficacy to manage one’s health—thereby enhancing the overall sexual well-being of survivors.

**Methods:**

Implemented at a Chinese tertiary hospital, this study adopted an exploratory sequential mixed-methods design. We first conducted in-depth qualitative interviews with 12 post-operative cervical cancer patients to investigate their sexual health experiences and unmet needs. Guided by these findings, a social media-based intervention was developed and delivered on WeChat, integrating three key elements: interactive multimedia education, a moderated peer support community, and specialist counseling. The efficacy of this digital intervention was then rigorously tested in a randomized controlled trial with 92 participants.

**Results:**

The qualitative interviews (*n* = 12) revealed five primary themes: physiological and psychological impacts, dynamic shifts in partnership, evolving self-perception, gaps in professional support, and conflicting expectations. These findings constructed a framework for a social media-based intervention. The subsequent randomized controlled trial (*n* = 92) demonstrated that the intervention group achieved a 19.08% increase in the total Female Sexual Function Index (FSFI) score at the 3-month follow-up (*p* < 0.001), with the most substantial improvement observed in sexual satisfaction (+49.79%). Notably, the intervention also led to a significant 13.97% increase in health empowerment (FACT-Cx, *p* < 0.001) and a 20.19% reduction in stigma (SIS, *p* < 0.001). Importantly, improvements in sexual function were strongly correlated with gains in health empowerment and reductions in stigma (*p* < 0.001).

**Conclusion:**

This digitally-enabled intervention bridges the principles of accessible public health communication with patient-centered care. By leveraging a widely-used social media platform, we delivered a holistic program that significantly enhanced sexual function, empowered patients, and mitigated stigma. This work establishes a practical, transferable solution for improving health literacy and quality of life among cancer survivors, demonstrating the potential to bridge service gaps in supportive care.

## 1 Introduction

Cervical cancer ranks among the most common gynecological malignancies affecting women worldwide (1). Radical hysterectomy (RHC) is the standard curative treatment for early-stage cervical cancer, significantly improving long-term survival rates (2). However, the extensive nature of this surgery often leads to pelvic autonomic nerve damage, which in turn triggers a series of postoperative complications. Among these, sexual dysfunction is particularly prevalent and carries profound long-term consequences (2, 3). Research indicates that postoperative patients frequently face challenges such as vaginal dryness, dyspareunia, decreased libido, and orgasmic difficulties, severely compromising their sexual quality of life (4, 5). Moreover, beyond the physical symptoms, this dysfunction is often shrouded in shame, embarrassment, and social stigma. Patients frequently hesitate to discuss these issues with their partners or healthcare providers due to cultural taboos and the fear of being judged, creating a significant ‘communication barrier’ that exacerbates their distress and isolation (6). Given the increasing emphasis on the long-term quality of life for cancer survivors and the explicit mandate within China’s “Healthy China Initiative” to enhance the quality of life for cancer patients, addressing the sexual health of cervical cancer survivors has become an urgent priority in clinical care (7).

Despite the high prevalence of sexual dysfunction following radical hysterectomy for cervical cancer, this issue is frequently overlooked in clinical practice due to constraints such as traditional cultural taboos, patient shame, and limited healthcare resources. This often leaves patients struggling to obtain effective professional guidance through routine follow-ups, resulting in a significant “information support gap” and “communication barrier” (8–10). Concurrently, the widespread adoption of digital technology and social media presents a timely opportunity to bridge this gap. Leveraging their accessibility, anonymity, and robust interactive capabilities, these platforms can overcome spatiotemporal limitations, offering patients private and convenient channels for information access and emotional support, thereby demonstrating immense potential for improving health literacy and addressing this unmet need (11–13).

Current studies have confirmed the positive role of social media in supporting cancer patients and health promotion (12, 13). However, most existing interventions utilize social media merely as a tool for one-way information dissemination, lack a systematic theoretical framework, and a comprehensive intervention program specifically targeting the sexual health of cervical cancer survivors that deeply integrates interactive communication theory with nursing science remains a gap (14). To address this, our study innovatively integrates news communication theory with nursing science to develop and validate a multi-component, social media-based digital health intervention. This program is designed not only to provide information but also to build a supportive online community, addressing patients’ multifaceted needs across physiological, psychological, and socio-relational dimensions, ultimately aiming to empower patients and enhance their sexual quality of life and overall health well-being. Specifically, this intervention aimed to enhance patients’ functional health literacy regarding post-operative sexual health. In this context, health literacy is conceptualized not merely as information recall, but as the development of knowledge, skills, and self-efficacy necessary to effectively manage one’s health condition (15). Our primary mechanism for achieving this was through the promotion of health empowerment, as measured by the FACT-Cx scale, which captures key domains of health literacy such as self-management, proactive life attitude, and ability to obtain support.

## 2 Research methods

### 2.1 Study design

This study employed an exploratory sequential mixed-methods design conducted at a tertiary hospital in Wuxi City, Jiangsu Province, China. The research comprised two distinct phases: (1) an initial qualitative phase involving in-depth interviews to explore patient experiences and inform intervention development, followed by (2) a subsequent quantitative phase utilizing a randomized controlled trial (RCT) to evaluate the intervention’s efficacy. This design was chosen to ensure the developed intervention was deeply grounded in the real-world needs and perspectives of the target population. The study received ethical approval from the Institutional Review Board of the Affiliated Hospital of Jiangnan University (WXSY-YXLL-AF/SC-11/02.0), and all participants provided written informed consent.

### 2.2 Phase 1: qualitative interview

#### 2.2.1 Participants and data collection

A purposive sampling strategy was employed to recruit participants from a tertiary Grade A hospital in Wuxi, Jiangsu Province. The inclusion criteria were: (1) women aged 18 years or older; (2) having undergone a radical hysterectomy for cervical cancer (International Federation of Gynecology and Obstetrics Stage I-IIA) between 6 months and 2 years prior to the study; and (3) self-reported experiencing any form of sexual dysfunction post-surgery. Participants were identified and referred by their attending physicians from the Gynecologic Oncology Department, after which the research team contacted them to explain the study and assess their interest in participation. A total of 12 patients were enrolled.

Semi-structured, face-to-face interviews were conducted by the first author in a private consultation room at the hospital. Each interview lasted approximately 30 to 60 min. The interview guide included open-ended questions focusing on patients’ physical and psychological experiences, changes in partnership dynamics, shifts in self-perception, and their needs and barriers concerning post-operative sexual health support. Data collection continued until data saturation was reached, which was defined as the point at which three consecutive interviews yielded no new themes or information. The detailed preparatory procedures for the interviews are illustrated in Supplementary Figure 1. The formal interview outline determined in this study is shown in Supplementary Figure 2.

#### 2.2.2 Data analysis

All interviews were audio-recorded, transcribed verbatim, and analyzed using the Colaizzi’s seven-step phenomenological analysis method. To enhance rigor, two researchers independently coded the first three transcripts. Both researchers were female graduate students in health communication. While they were not survivors of cervical cancer, they received extensive training in qualitative interviewing and sensitivity to the topic. Their potential bias was an assumption of significant patient distress, which was mitigated by using open-ended questions and allowing themes to emerge from the data. After independent coding, the researchers met to compare their initial codes and develop a preliminary codebook. Any discrepancies in coding were discussed until a consensus was reached. The inter-coder agreement rate before consensus was approximately 85%. The remaining transcripts were then coded by the first author using the finalized codebook, with regular discussions held with the corresponding author to review and refine the emerging themes.

#### 2.2.3 Intervention development informed by qualitative findings

Thematic analysis of the qualitative interviews identified five key themes: physiological and psychological impacts, dynamic remodeling of partnerships, shifts in self-perception, gaps in sexual health support, and conflicting expectations. These findings collectively highlighted a critical “information support gap” and profound “communication barriers,” underscoring the need for a multi-faceted support platform.

Guided by these patient-centered insights and grounded in Nutbeam’s hierarchy of health literacy (16), which conceptualizes health literacy progression from functional to interactive and finally to critical levels, we designed a comprehensive WeChat-based digital health intervention. The 5-week program was structured to systematically address these different levels of health literacy through three core, interconnected components.

##### 2.2.3.1 Interactive multimedia education to build functional health literacy

This component was designed to develop functional health literacy by providing accessible, understandable information on post-operative sexual health. A series of short videos, infographics, and articles were distributed weekly via the private WeChat group. The content covered essential knowledge, including anatomical changes post-hysterectomy, practical symptom management (e.g., for vaginal dryness and dyspareunia), communication techniques for discussing issues with partners, and strategies for psychological adaptation. By delivering this content in a private, multimedia format, we aimed to equip participants with the foundational knowledge and skills to understand and manage their condition, directly addressing the identified “information support gap.”

##### 2.2.3.2 Moderated peer support community to foster interactive health literacy

To advance health literacy to the interactive level, which involves the development of social and cognitive skills to extract information and derive meaning from different forms of communication, we established a dedicated, anonymized WeChat group facilitated by a trained research nurse. This community provided a secure environment for participants to share personal experiences, seek and offer informal advice, and provide mutual encouragement. This interactive dialogue fostered not only emotional support but also enabled participants to apply knowledge in a social context, refine their communication skills, and critically engage with peer experiences, thereby breaking down the “communication barriers” identified in our qualitative work.

##### 2.2.3.3 Clinical specialist-led counselling to promote critical health literacy

The final component aimed to cultivate critical health literacy, the highest level, which entails the ability to critically analyze information and exert greater control over life events and situational determinants of health. Scheduled live Q&A sessions were conducted within the WeChat group by gynecological oncology clinical specialists and certified sexual health counselors. These sessions provided authoritative expertise and personalized guidance, empowering participants to make informed decisions, challenge misconceptions, and advocate for their own needs. This direct access to experts bridged the gap in professional support and was instrumental in enhancing participants’ self-efficacy and empowerment, enabling them to take greater control over their health and well-being.

The selection of WeChat as the delivery platform was strategic, leveraging its ubiquitous use, multimedia capabilities, and features for both private consumption of information (e.g., one-way push of educational materials) and dynamic social interaction (e.g., group chats and live sessions). This ensured the intervention was not only theoretically sound but also highly accessible and ecologically valid for the target population. A detailed mapping of theoretical constructs to intervention components is provided in Supplementary Table 11.

### 2.3 Phase 2: quantitative RCT study

#### 2.3.1 Participants and recruitment

A convenience sample was recruited from the gynecological oncology outpatient clinic of a tertiary hospital in Wuxi, China. Participants were screened against the following criteria.

##### 2.3.1.1 Inclusion criteria

Patients diagnosed with stage I–II cervical cancer according to FIGO (International Federation of Gynecology and Obstetrics) criteria; aged between 18 and 50 years; had undergone radical hysterectomy and had been postoperative for more than 6 months; were in a stable partnership, defined as being married or cohabiting with a partner for at least 6 months, and had a history of sexual activity; had clear consciousness and normal speech expression ability; voluntarily participated in this study and provided informed consent.

##### 2.3.1.2 Exclusion criteria

Patients with mental disorders, cognitive impairments, or other conditions that prevent normal communication; patients with cardiovascular or cerebrovascular diseases, or suffering from other tumors, which could affect sexual life; patients with sexual psychological disorders or sexual dysfunction prior to their cancer diagnosis; patients who, upon confirmation with their attending physician, were not fully informed of their cervical cancer diagnosis. This criterion addresses the occasional cultural practice where family members request the medical team to disclose a less severe diagnosis (e.g., “benign uterine tumor” or “severe inflammation”) to the patient as a protective measure, which would otherwise preclude obtaining fully informed consent for this cancer-specific study.

One hundred and eight patients were initially assessed for eligibility.

#### 2.3.2 Sample size estimation

An *a priori* sample size calculation was performed using G*Power software (Version 3.1). The calculation was based on the primary outcome, the Female Sexual Function Index (FSFI) total score, and the anticipated interaction effect in a repeated-measures analysis of variance (RM-ANOVA) between the two groups across three time points.

As this was an exploratory pilot randomized controlled trial, no prior directly comparable studies were available to precisely estimate the effect size. Therefore, we adopted a conservative approach by anticipating a medium-to-large effect size (*f* = 0.40) for the group × time interaction. This threshold is widely recognized in behavioral and health intervention research as a clinically meaningful and realistic target for novel interventions.

With an alpha (*α*) level set at 0.05 and a desired power (1 − *β*) of 0.80, the analysis indicated a required total sample size of 84 participants (42 per group). To account for an estimated attrition rate of approximately 20% over the study period, we aimed to recruit a total of 100 participants (50 per group).

#### 2.3.3 Randomization and procedure

Eligible participants were randomly assigned to either the intervention group or the control group using a computer-generated random sequence. The intervention group received the 5-week WeChat-based intervention in addition to routine care, while the control group received routine care only. Routine care consisted of standard postoperative follow-up visits scheduled at the hospital at 1, 3, 6, and 12 months, which primarily focused on monitoring for cancer recurrence through physical examination and did not include structured assessment, education, or counseling regarding sexual health. Control group participants were not provided with any additional digital resources or educational materials as part of this study. To minimize contamination risk, participants in both groups were explicitly instructed not to discuss the study’s specific intervention content with participants from the other group. Given that the intervention was delivered in a private, dedicated WeChat group, the risk of cross-group communication was considered low.

#### 2.3.4 Data collection and measures

Data were collected at baseline (T0), 1-month post-intervention (T1), and 3-months post-intervention (T2). The primary and secondary outcome measures were:

Female Sexual Function Index (FSFI): The primary outcome, a validated 19-item questionnaire assessing six domains of sexual function: desire, arousal, lubrication, orgasm, satisfaction, and pain. The total score ranges from 2.0 to 36.0, with a higher score indicating better sexual function. In the present sample, the Cronbach’s alpha for the FSFI total score at baseline was 0.88, indicating good internal consistency.
Functional Assessment of Cancer Therapy-Cervix (FACT-Cx): Used to measure health empowerment, focusing on self-management, life attitude, and social support. A higher score represents a greater level of health empowerment. In this study, the overall FACT-Cx scale demonstrated excellent internal consistency, with a Cronbach’s alpha of 0.85 at baseline.
Social Impact Scale (SIS): Given that the experience of sexual dysfunction is profoundly influenced by perceived stigma and social judgment—a key barrier identified in our qualitative phase—we used the Social Impact Scale to assess this construct. Higher scores reflect a higher level of perceived stigma. The internal consistency for the SIS in our sample at baseline was good, with a Cronbach’s alpha of 0.81.

#### 2.3.5 Statistical analysis

Data were analyzed using SPSS software. Descriptive statistics, independent samples t-tests, and Chi-square tests were used to compare baseline characteristics between groups. The normality of the distribution for continuous outcome variables (FSFI, FACT-Cx, SIS) was confirmed using the Shapiro–Wilk test (all *p* > 0.05), justifying the use of parametric tests. The primary analysis for evaluating intervention effects was conducted using Repeated-Measures Analysis of Variance (RM-ANOVA) to examine the interaction effects between group and time. Additionally, to robustly assess between-group differences at post-intervention timepoints while controlling for baseline scores, Analysis of Covariance (ANCOVA) was performed on the 1-month (T1) and 3-month (T2) outcomes, with the respective baseline (T0) scores as the covariate. Pearson correlation analysis was conducted to examine the relationships between FSFI, FACT-Cx, and SIS scores. A two-sided *p*-value of less than 0.05 was considered statistically significant. Effect sizes were calculated and reported for all between-group comparisons. Cohen’s *d* was used for *t*-test comparisons, with interpretations based on conventional thresholds: small (*d* = 0.2), medium (*d* = 0.5), and large (*d* = 0.8). Partial *η*^2^ was used for ANOVA and ANCOVA analyses.

#### 2.3.6 Intervention fidelity and adherence

Intervention fidelity and participant engagement were systematically monitored throughout the 5-week program. Overall, the intervention was implemented as planned, and participant adherence was satisfactory, with patterns of engagement reflecting the practical challenges of a real-world digital health study.

##### 2.3.6.1 Interactive multimedia education

The weekly educational materials were successfully delivered to all 46 participants in the intervention group. Engagement was high but not uniform. The view rate for the weekly modules was highest in the first week (42 participants, 91.3%) and saw a slight decline by the fourth week (36 participants, 78.3%), consistent with the natural attrition of engagement in longitudinal digital interventions.

##### 2.3.6.2 Moderated peer support community

The WeChat group remained active for the duration of the study. A total of 408 messages were exchanged. Engagement was distributed unevenly, as is common in peer support communities. Approximately 65% of participants (*n* = 30) posted at least one message or question, while the remaining 35% (*n* = 16) were predominantly passive observers (“lurkers”) who, according to post-study feedback, still found value in reading the shared experiences.

##### 2.3.6.3 Clinical specialist-led counselling

Two live Q&A sessions were held. The first session had an attendance of 37 participants (80.4%), and the second session was attended by 33 participants (71.7%). For those unable to attend, the text summaries and key takeaways from the sessions were posted in the group the following day, and were viewed by an additional 8 participants.

In summary, the digital intervention was delivered with high fidelity to the protocol. While participant adherence naturally varied, the overall engagement metrics confirm that the majority of participants were actively involved with the core components, supporting the feasibility and acceptability of the program.

## 3 Results

### 3.1 Qualitative findings: co-existing scarcity of medical resources and communication barriers

**Figure 1:**
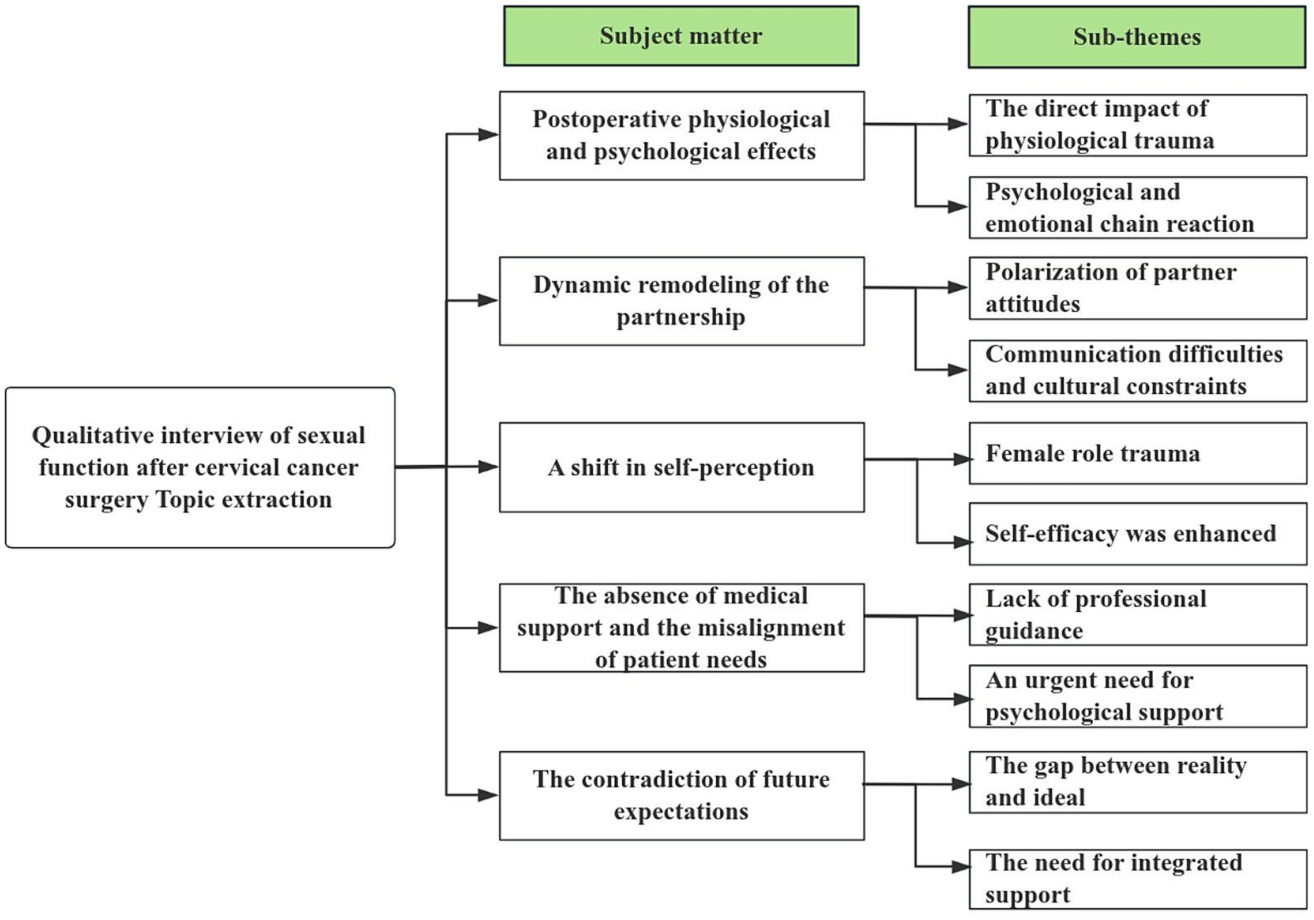
Qualitative interview research themes.

Our initial in-depth qualitative interviews with 12 cervical cancer survivors experiencing sexual dysfunction post-radical hysterectomy provided profound insights into their lived experiences. Thematic analysis of the interview data culminated in five core themes and several sub-themes, which are visually summarized in Figure 1. Below, we present these themes, illustrated with anonymized verbatim excerpts from the participants to ground the findings in their authentic voices.

#### 3.1.1 Theme 1: the multifaceted impact of postoperative physiological and psychological effects

Patients reported a complex interplay between physical sequelae and emotional distress. This theme encompasses the direct physical trauma and its subsequent psychological ripple effects.

Sub-theme: The direct impact of physiological trauma: Participants frequently described specific physical challenges that directly hindered sexual activity. *“It feels shorter and tighter than before. There’s often dryness and pain during intercourse, which makes me very fearful.”* (Participant 3). Another stated, *“I barely have any sexual desire now. It’s like that part of me is gone.”* (Participant 7).

Sub-theme: Psychological and emotional chain reaction: The physical changes triggered significant emotional suffering. *“After the surgery, I feel incomplete, less of a woman. This thought lingers and affects my mood every day.”* (Participant 10). Another participant shared, *“I feel anxious and depressed. I’m afraid my husband will be disappointed, so I sometimes make excuses to avoid intimacy.”* (Participant 5).

#### 3.1.2 Theme 2: dynamic remodeling of the partnership

The changes in sexual function necessitated a renegotiation of intimacy and roles within the couple, with partners’ attitudes ranging from supportive to distancing.

Sub-theme: Polarization of partner attitudes: Experiences with partners varied greatly. Some participants felt supported: *“My husband is very understanding. He says it does not matter and that health is the most important thing.”* (Participant 2). Others felt a growing distance: *“He says it’s okay, but I can feel him being more cautious, and we do not talk about it anymore. It’s like there’s a wall between us now.”* (Participant 8).

Sub-theme: Communication difficulties and cultural constraints: Many couples struggled to discuss the issue openly. *“We Chinese do not easily talk about these things in bed. It’s too embarrassing. So we both pretend nothing is wrong, but the problem is there.”* (Participant 11).

#### 3.1.3 Theme 3: a shift in self-perception

The experience of cancer and its treatment led to a profound re-evaluation of self-identity and femininity, which could be both traumatic and empowering.

Sub-theme: Female role trauma: The loss of reproductive capacity and sexual function deeply impacted self-identity. *“A woman’s uterus is so important. After removing it, I feel like I’m no longer a complete woman. I’ve even lost confidence in social situations.”* (Participant 9).

Sub-theme: Self-efficacy was enhanced (in some individuals): Conversely, a few participants found new strength. *“Going through this made me realize how strong I am. I’m actively looking for information and trying different ways to improve the situation. I have to take charge of my own life.”* (Participant 4).

#### 3.1.4 Theme 4: the absence of medical support and the misalignment of patient needs

A prominent theme was the stark gap between the support patients needed and what they received from the healthcare system.

Sub-theme: Lack of professional guidance: Participants universally reported a lack of actionable advice. *“The doctor just said ‘you can live a normal life after three months’ and that was it. What is a ‘normal life’? How to deal with the pain? There was no guidance.”* (Participant 1).

Sub-theme: An urgent need for psychological support: The need extended beyond physical solutions. *“I wish there was a channel where I could talk about my psychological struggles. The follow-ups only check for cancer recurrence, but nobody asks about my mental health or sexual life.”* (Participant 6).

#### 3.1.5 Theme 5: the contradiction of future expectations

Patients grappled with conflicting feelings about the future, torn between hope for improvement and the fear of permanent loss.

Sub-theme: The gap between reality and ideal: There was a painful awareness of the disparity between their current state and pre-illness normality. *“I know it will probably never be the same as before I got sick. I have to accept that, but it’s really hard.”* (Participant 12).

Sub-theme: The need for integrated support: Participants expressed a strong desire for holistic, ongoing support. *“We need more than just a one-time consultation. We need continuous support, including knowledge, psychological counseling, and communication with people who have the same experience.”* (Participant 2).

These findings clearly demonstrate that traditional clinical communication channels failed to meet patients’ needs for private, empathetic, and continuous support regarding sexual health (17, 18). The expressed needs for confidential information, peer understanding, professional guidance, and holistic support directly informed the design of our social media-based intervention, positioning it as a digital solution to bridge this critical sexual health communication dilemma.

### 3.2 Quantitative outcomes: efficacy of the social media-based intervention

#### 3.2.1 Participant flow and baseline characteristics

**Figure 2:**
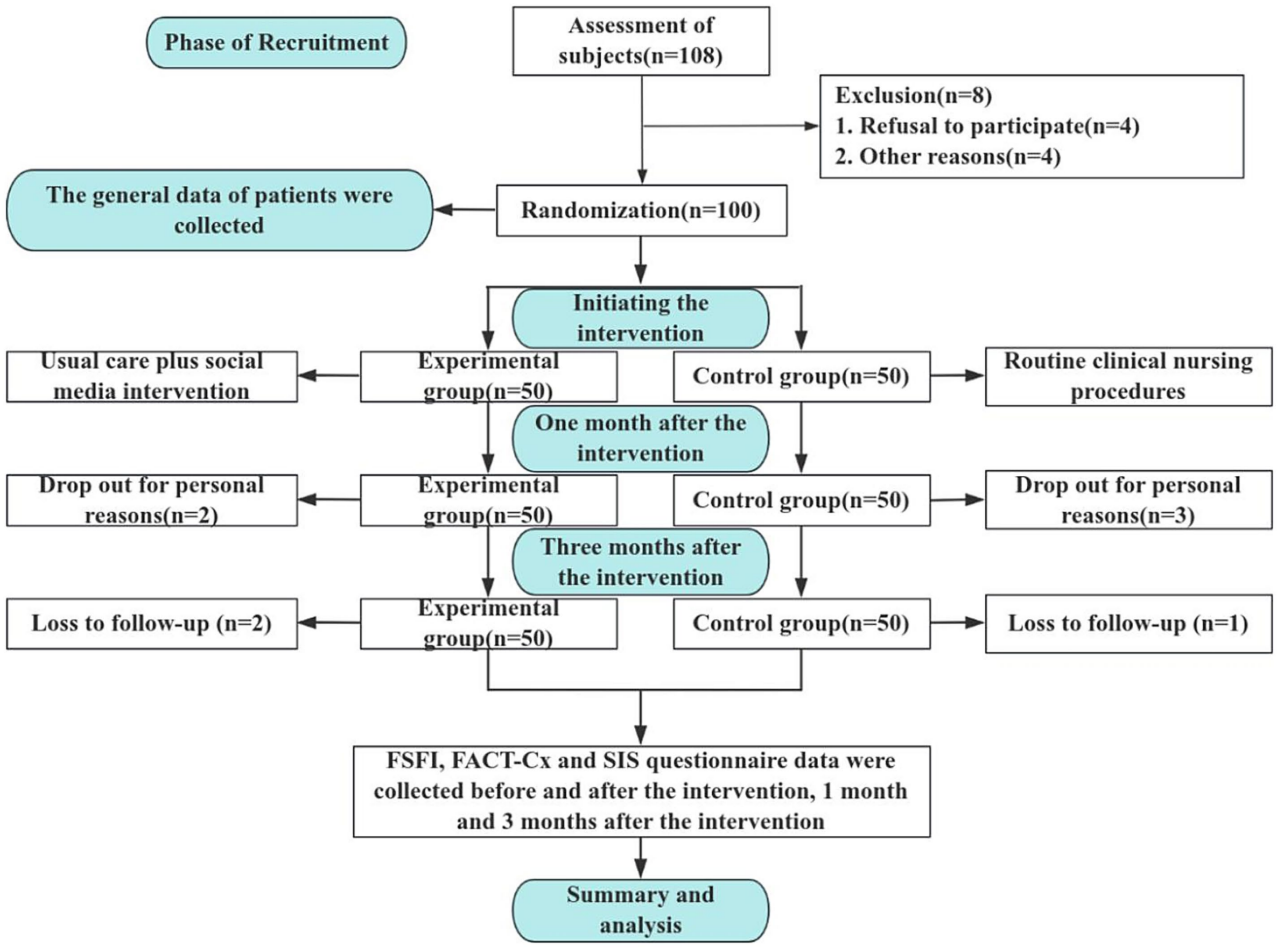
Flow chart of enrolled patients.

As shown in the CONSORT flow diagram (Figure 2), 100 patients were initially enrolled in the clinical trial. During the trial, 5 participants withdrew due to personal reasons, and 3 were lost to follow-up. Consequently, 92 participants completed the randomized controlled trial, with 46 allocated to each the intervention and control groups. No statistically significant differences in demographic or clinical characteristics were observed at baseline (*p* > 0.05, Supplementary Table 1), ensuring comparability between the groups.

#### 3.2.2 Primary outcome: improvement in sexual function

The intervention group demonstrated significant and sustained improvements in sexual function, as measured by the Female Sexual Function Index (FSFI). While no inter-group differences were observed at baseline (*p* > 0.05, Supplementary Table 2), the intervention group exhibited significantly higher total FSFI scores at both the 1-month (*p* < 0.001, Cohen’s *d* = 0.71, medium-to-large effect) and 3-month follow-ups (*p* < 0.001, Cohen’s *d* = 1.95, very large effect; Supplementary Tables 3, 4). The magnitude of improvement increased over time, with particularly strong effects observed for sexual desire (*d* = 1.16) and sexual satisfaction (*d* = 0.98) at 3 months. The RM-ANOVA revealed a significant group × time interaction effect for the total FSFI score (*F*(2, 180) = 25.84, *p* < 0.001, partial *η*^2^ = 0.223). Confirming this finding, the ANCOVA models controlling for baseline scores demonstrated that the intervention group had significantly higher FSFI total scores than the control group at both the 1-month (*F*(1, 89) = 14.80, *p* < 0.001) and 3-month follow-ups (F(1, 89) = 53.44, *p* < 0.001) (For detailed ANCOVA results of all outcomes, see Supplementary Table 10).

**Figure 3:**
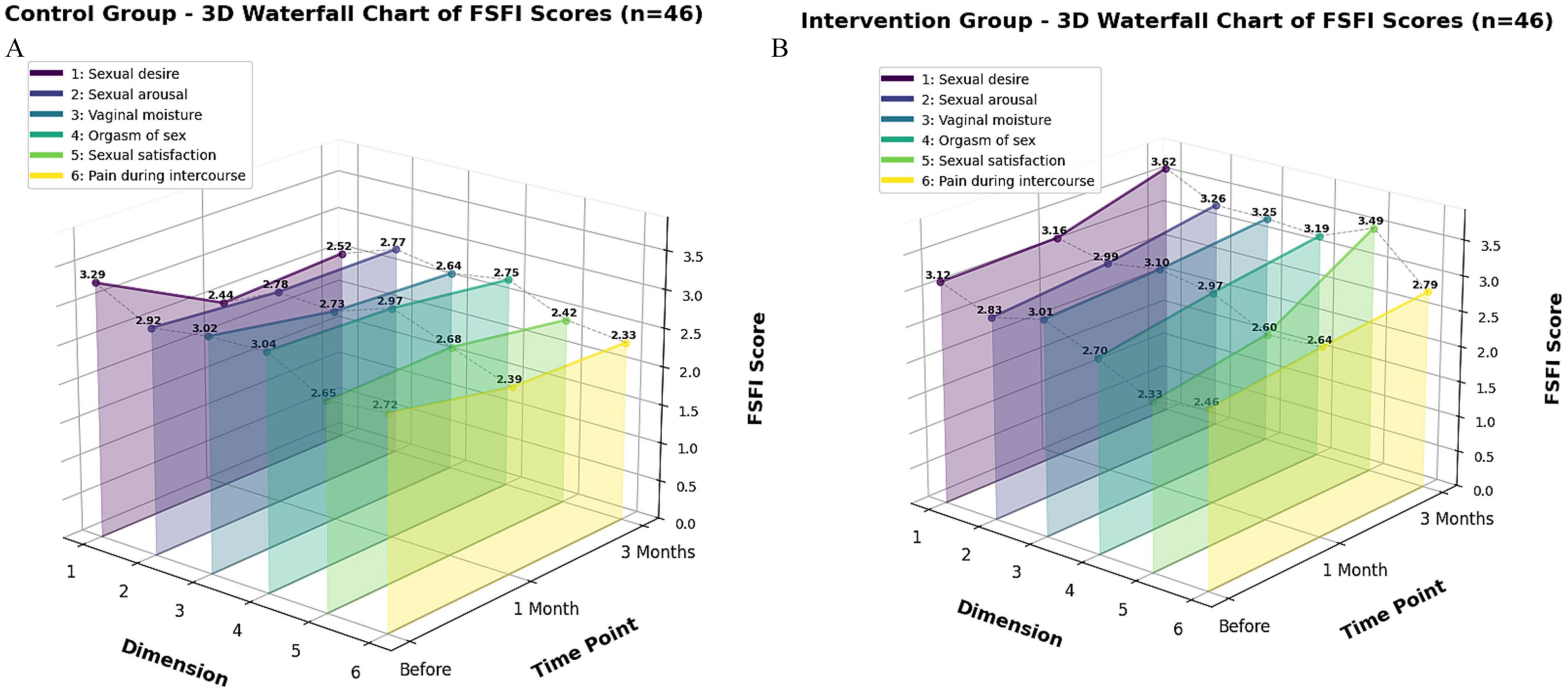
**(A,B)** Waterfall chart of the changes in the scores of the six dimensions of the FSFI scale between the control group and the intervention group. Panel **A** shows the changes in the scores of the six dimensions of the FSFI scale in the control group before and after the intervention, and panel **B** shows the changes in the scores of the six dimensions of the FSFI scale in the intervention group before and after the intervention. The Z-axis represents the time points of the control, which are before the intervention, 1 month after the intervention, and 3 months after the intervention; the X-axis corresponds to the six dimensions of the FSFI scale, where 1 (dark purple) is the score of sexual desire, 2 (blue purple) is the score of sexual arousal, 3 (blue) is the score of vaginal moisture, 4 (dark green) is the score of orgasm of sex, 5 (light green) is the score of sexual satisfaction, and 6 (light yellow) is the score of pain during intercourse; the Y-axis represents the average score of each dimension, and the higher the score, the better the sexual function of the patients in that dimension.

**Figure 4:**
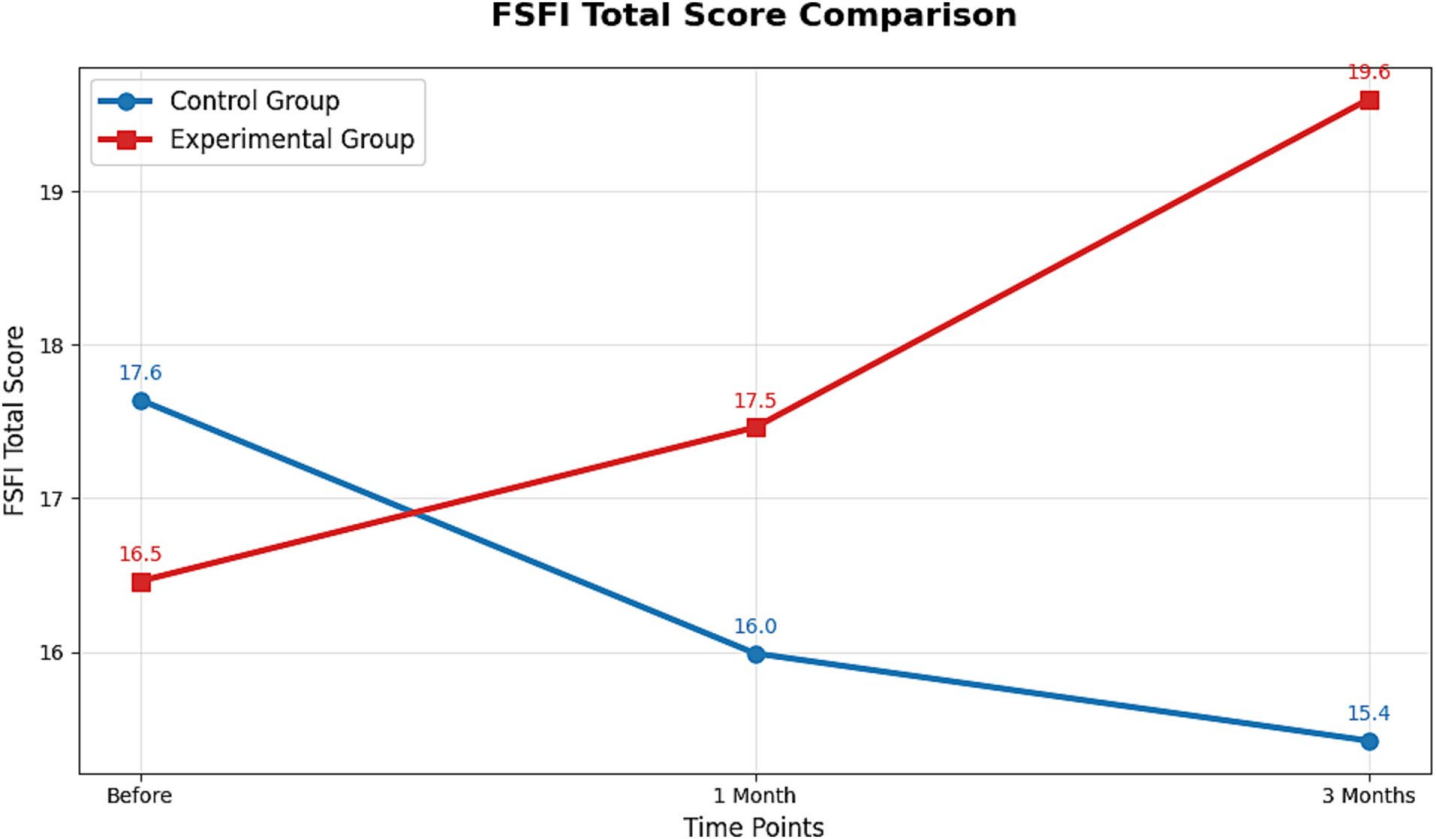
Line graphs of the average score changes of the FSFI scale before and after the intervention in the control group and the intervention group. This figure shows the comparison of the total scores of the FSFI scale before and after the intervention in the control group and the intervention group. The X-axis represents the control time points, which are before the intervention, 1 month after the intervention, and 3 months after the intervention. The Y-axis represents the average total score of the FSFI scale. The higher the score, the better the sexual function status of the patients in that group. The blue line represents the control group, and the red line represents the intervention group.

The 3D waterfall plots in Figure 3A (control group) and Figure 3B (intervention group) visually depict the distinct trajectories of the six FSFI subdomain scores between the groups, highlighting the multi-faceted nature of the improvement. In terms of the magnitude of change, the total FSFI score in the intervention group increased by 6.08% at 1 month and 19.08% at 3 months, whereas a decrease was observed in the control group (Supplementary Table 5). This overall trend is clearly illustrated in the line graph (Figure 4). The most remarkable improvement was noted in the domain of sexual satisfaction, which surged by 49.79% in the intervention group at the 3-month mark (Supplementary Table 5, and visually evident in Figure 3B).

#### 3.2.3 Secondary outcomes: enhanced health empowerment and reduced stigma

**Figure 5:**
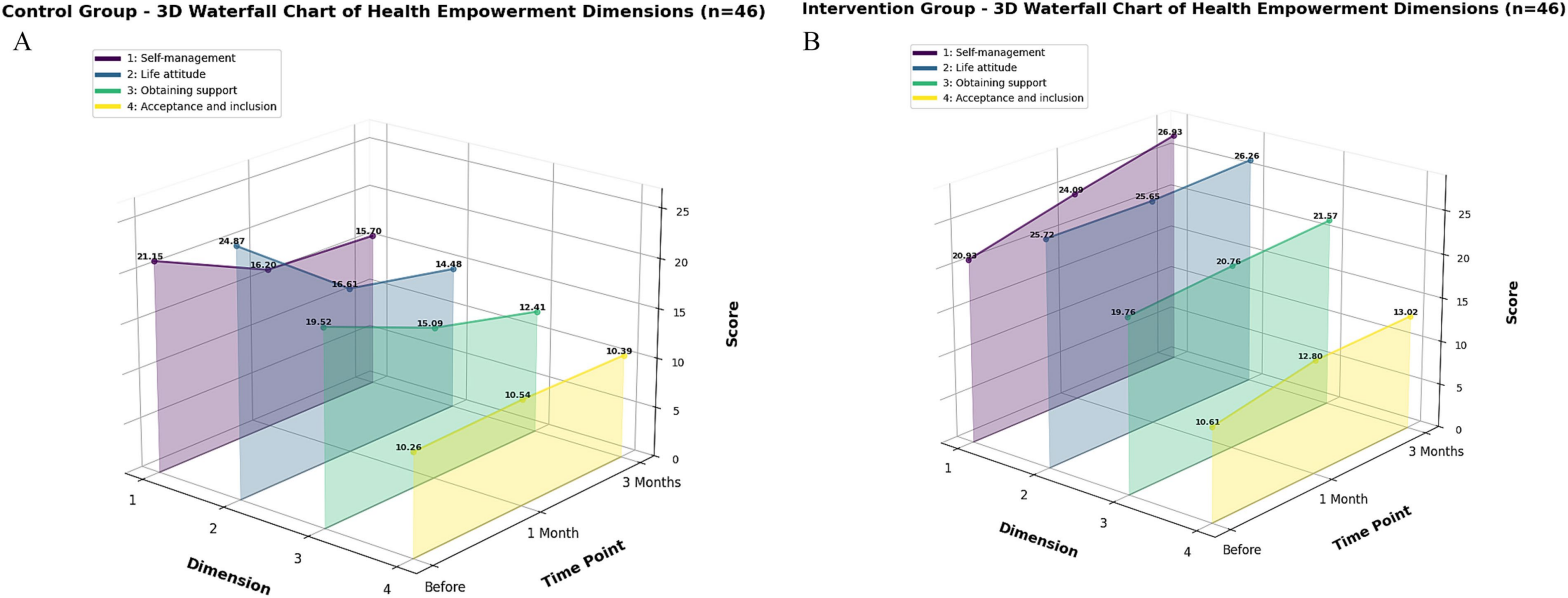
**(A,B)** Waterfall chart of the changes in the scores of the four dimensions of the FACT-Cx scale between the control group and the intervention group. Panel **A** shows the changes in the scores of the four dimensions of the FACT-Cx scale in the control group before and after the intervention, while panel **B** shows the changes in the scores of the four dimensions of the FACT-Cx scale in the intervention group before and after the intervention (1 month and 3 months after the intervention). The Z-axis represents the control time points, which are before the intervention, 1 month after the intervention, and 3 months after the intervention; the X-axis corresponds to the four dimensions of the FACT-Cx scale, where 1 (purple) is the self-management score, 2 (blue) is the life attitude score, 3 (green) is the obtaining support score, and 4 (yellow) is the acceptance and inclusion score; the Y-axis represents the average score of each dimension, with higher scores indicating better conditions for the patients in that dimension.

**Figure 6:**
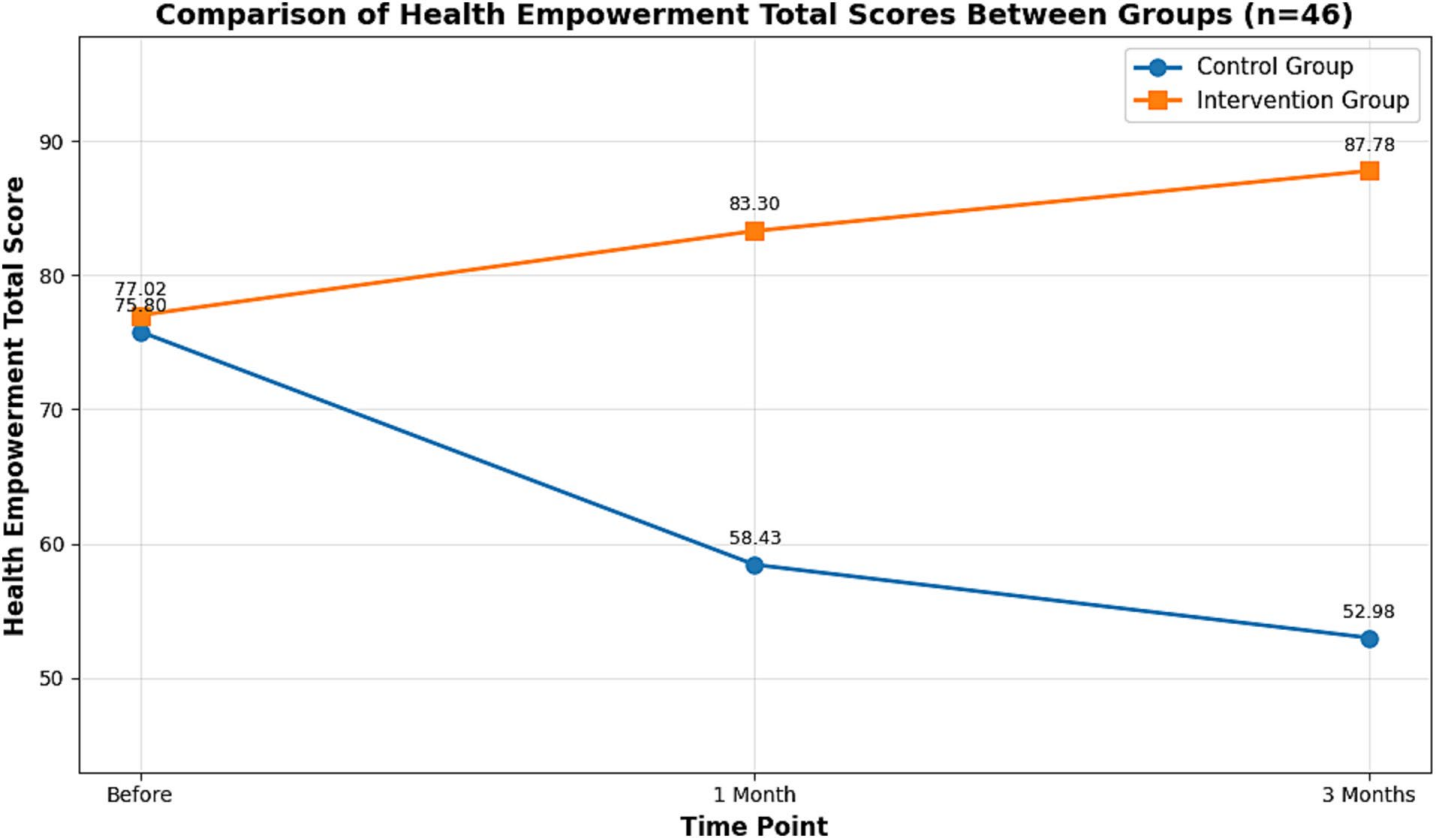
Line graphs of the average score changes of the FACT-Cx scale before and after the intervention in the control group and the intervention group. This figure shows the comparison chart of the health empowerment ability (FACT-Cx scale) scores of the patients before and after the intervention. The X-axis represents the time points, which are before the intervention, 1 month after the intervention, and 3 months after the intervention; the Y-axis represents the average score of this group of patients. The blue line represents the control group and the orange line represents the intervention group.

The impact of the intervention extended beyond sexual function with substantial effect sizes. On the FACT-Cx scale, the intervention group showed large increases in overall health empowerment at both 1-month (Cohen’s *d* = 1.88) and 3-month follow-ups (Cohen’s *d* = 2.07; Supplementary Tables 7, 8). Particularly notable were the very large improvements in self-management (*d* = 2.68 at 3 months) and life attitude (*d* = 2.39 at 3 months). Concurrently, perceived stigma was significantly reduced with a large effect size (*d* = 1.41 at 3 months). The ANCOVA results, adjusting for baseline scores, further validated these findings, showing significant between-group differences in health empowerment (FACT-Cx) and stigma (SIS) at both post-intervention assessments (all *p* < 0.001), with effect sizes ranging from partial *η*^2^ = 0.071 to 0.920 (see Supplementary Table 10). Specifically, the intervention group achieved a 13.97% increase in overall health empowerment at 3 months (*p* < 0.001). The 3D waterfall plots in Figure 5A (control group) and Figure 5B (intervention group) vividly illustrate the contrasting changes in the four subscales of health empowerment, with the self-management subscale demonstrating a particularly striking improvement of 28.67% (Supplementary Tables 6–9). The progression of the total health empowerment scores for both groups is further detailed in the line graph (Figure 6).

**Figure 7:**
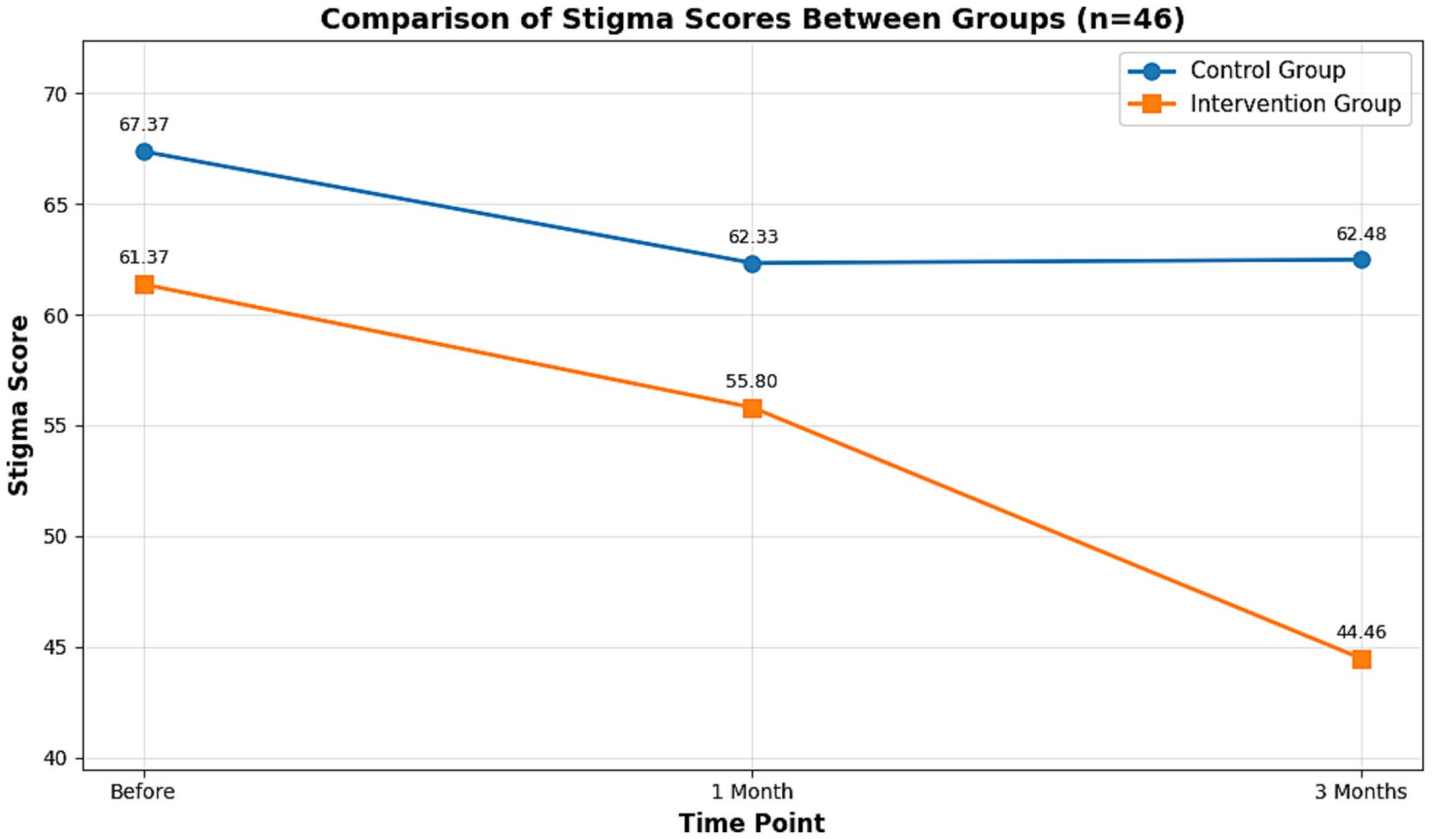
Line graphs of the average score changes of the SIS scale before and after the intervention in the control group and the intervention group. This figure shows the comparison chart of the patients’ stigma scores (based on the SIS scale) before and after the intervention. The lower the score, the lower the degree of stigma for the patients. The X-axis represents the time points, which are before the intervention, 1 month after the intervention, and 3 months after the intervention; the Y-axis represents the average score of this group of patients, with blue representing the control group and orange representing the intervention group.

Concurrently, perceived stigma, measured by the SIS, was significantly reduced by 20.19% in the intervention group at 3 months (*p* < 0.001), while it remained largely unchanged in the control group (Supplementary Tables 6–9). This divergent trend in stigma reduction is clearly captured in the line graph presented in Figure 7.

#### 3.2.4 Correlation analysis: sexual health strongly correlates with stigma and health empowerment

**Figure 8:**
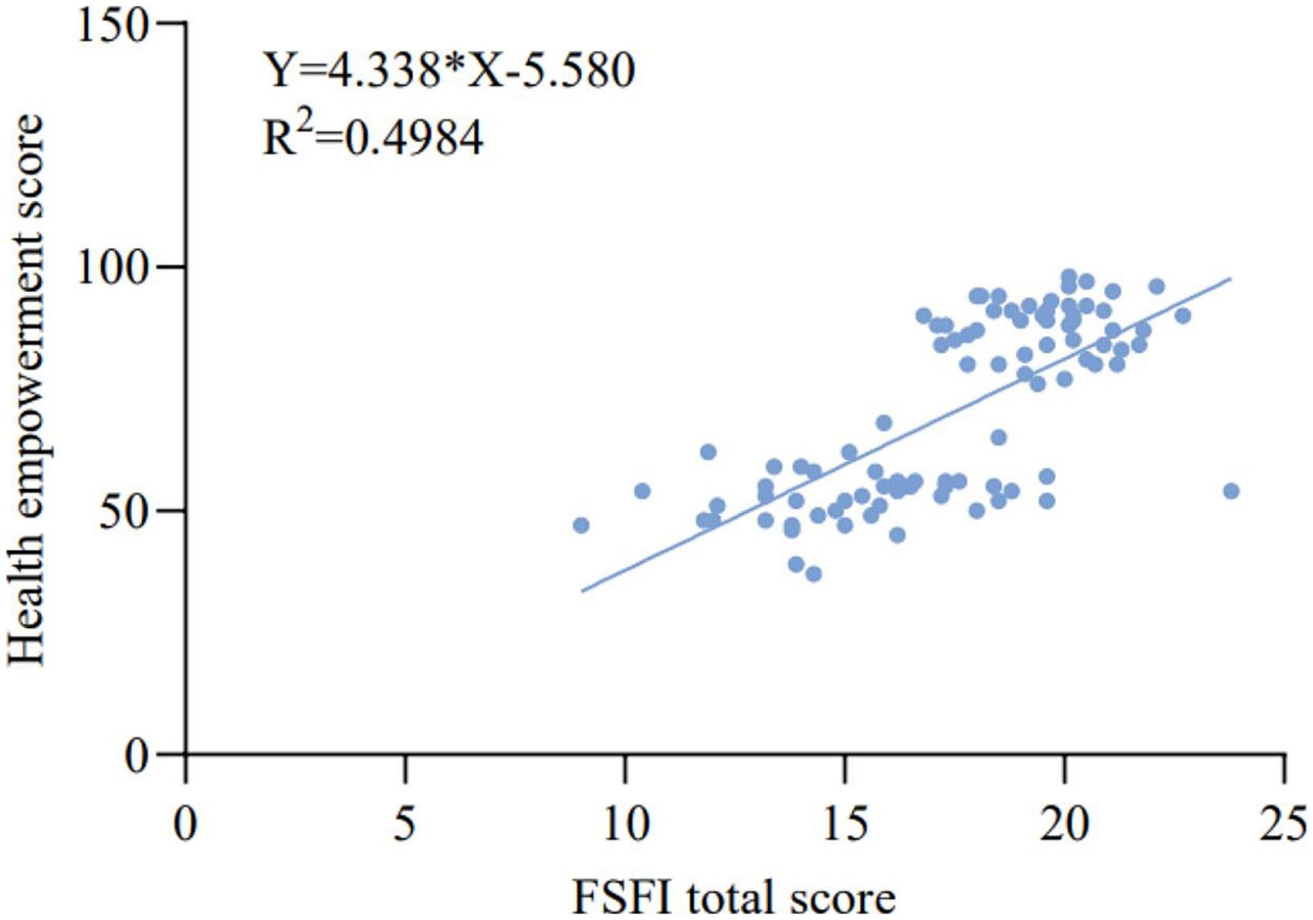
Correlation between FSFI total score and health empowerment. This figure shows whether there is a correlation between the FSFI scale (indicating the improvement in sexual function) and the FACT-Cx scale (indicating health empowerment) in the patients after the intervention. Therefore, this study extracted the data 3 months after the intervention for correlation analysis. The X-axis represents the total score of the FSFI scale, and the Y-axis represents the total score of the FACT-Cx health empowerment. After data analysis, *p* < 0.001, and the scatter plot shows a positive correlation. Thus, patients with higher health empowerment ability have better sexual function improvement.

**Figure 9:**
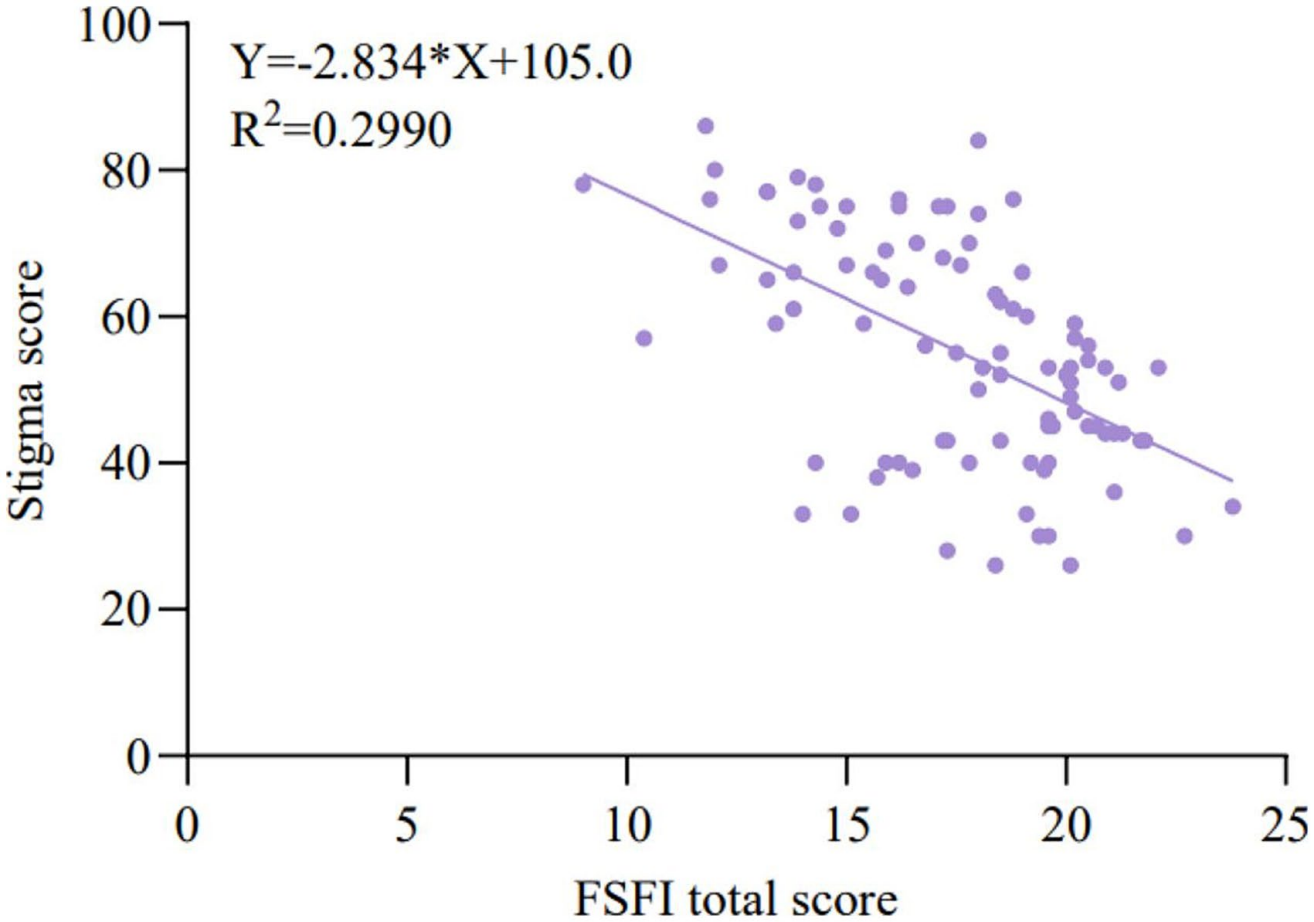
Correlation between FSFI total score and perceived stigma. This figure shows whether there is a correlation between the FSFI scale (indicating the improvement in sexual function) and the SIS scale (indicating the sense of stigma) in the patients after the intervention. Therefore, this study extracted the data 3 months after the intervention for correlation analysis. The X-axis represents the total score of the FSFI scale, and the Y-axis represents the total score of the SIS scale for the sense of stigma. After data analysis, *p* < 0.001, and the scatter plot shows a negative correlation. Therefore, the lower the score of the sense of stigma (the lower the degree of stigma in the patients), the better the improvement in sexual function.

Pearson correlation analysis revealed a strong positive correlation between the total FSFI score and health empowerment (*p* < 0.001, Figure 8), and a strong negative correlation between the total FSFI score and stigma (*p* < 0.001, Figure 9). This indicates that improvements in sexual function were closely associated with enhanced patient confidence in managing their health and a reduction in feelings of shame.

## 4 Discussion

Our findings validate the efficacy of this digitally-enabled intervention in bridging a critical support gap in the sexual health of cervical cancer survivors. By comparing against a control group that received only routine care (i.e., lacking structured sexual health guidance), this study demonstrates that a private digital ecosystem created on the ubiquitous WeChat platform successfully circumvented the dual challenges of cultural taboo and clinical resource limitations. It delivered a sustainable, participant-centered model that significantly enhanced sexual function, promoted health literacy, and empowered patients by mitigating stigma.

### 4.1 Bridging the digital health communication gap

The qualitative findings underscore a stark reality: a “systemic silence” persists in clinical practice regarding sexual dysfunction, despite its high prevalence (19, 20). Patients desire information but lack private, reliable, and continuous access channels. Our WeChat-based intervention was fundamentally designed to break this silence. Guided by Nutbeam’s hierarchy of health literacy, our program was structured to progress from foundational knowledge to critical empowerment (16). The interactive multimedia education component served as a confidential source of authoritative knowledge, directly enhancing participants’ functional health literacy by making complex information accessible and comprehensible. The moderated peer support community fostered a safe space for knowledge sharing and emotional support, effectively developing interactive health literacy through lived experience, targeted learning, and social communication. Most importantly, the significant improvements observed in health empowerment (FACT-Cx), particularly in self-management and life attitude, signify the development of critical health literacy—equipping patients with the confidence and skills to exert greater control over their health and well-being. This multi-faceted approach exemplifies how digital communication can be tailored to overcome information barriers and foster empowerment among vulnerable populations, a key insight of this study (21, 22).

### 4.2 Empowering patients beyond sexual function

The significant improvements in FSFI scores, particularly the 49.79% surge in sexual satisfaction, validate the intervention’s efficacy. More importantly, the marked increase in FACT-Cx and the reduction in SIS reveal a deeper impact. The intervention did not merely provide information; it equipped patients with the confidence (self-efficacy), skills (self-management), and a supportive, anonymous online community to actively manage their health and well-being. The strong correlations among FSFI, empowerment, and stigma suggest a virtuous cycle: as patients become more knowledgeable and empowered, they experience less shame, are more likely to engage in and enjoy sexual activity, which in turn further reinforces their health empowerment (23, 24). This aligns perfectly with the goals of digital health communication to support informed decision-making and foster healthy lifestyles.

### 4.3 Cultural context and implications

Our findings must be interpreted within the Chinese cultural context, where sexuality is often a taboo subject, and open discussion, especially for women, is constrained by traditional norms. This ‘culture of silence’ amplifies the stigma and isolation experienced by survivors (6, 20). The significant reduction in stigma observed in our intervention group is therefore particularly noteworthy. The anonymity afforded by the digital platform likely provided a “safe space” that circumvented cultural barriers to communication. Participants could seek information and share experiences without the fear of “losing face” or being judged, which is difficult to achieve in traditional face-to-face clinical settings in this cultural context. While the specific content (e.g., communication techniques) might require cultural adaptation, the core model—leveraging a ubiquitous, private digital platform to break the silence and provide holistic support—holds great promise for transferability to other cultural settings with similar taboos around female sexuality.

### 4.4 A scalable and participatory digital public health model

Unlike interventions that use social media merely as a one-way information delivery tool, our program emphasized participatory engagement. The peer support group and live Q&A sessions created dynamic, multi-directional communication, engaging patients as active participants in their own care rather than passive recipients of information. This model, centered on a platform already integrated into daily life, provides a scalable, cost-effective, and highly accessible solution. It reduces the workload for healthcare professionals while extending the reach of care beyond the hospital walls, directly addressing issues of accessibility and equity (25).

### 4.5 Limitations and future directions

This study has several limitations that should be acknowledged when interpreting the results. First, the single-center design may limit the generalizability of our findings to other healthcare settings or patient populations with different cultural or socioeconomic backgrounds. Second, the relatively short 3-month follow-up period, while demonstrating significant short-term efficacy, is insufficient to determine the long-term sustainability of the intervention’s effects, as sexual recovery is a continuous process that often extends beyond 12 months. Third, the absence of the partners’ perspectives represents a critical limitation, as their attitudes, sexual function, and communication skills are integral to dyadic sexual recovery and relational outcomes; our patient-centric focus, while providing initial efficacy data, omits this crucial dimension.

Methodologically, our study is subject to several potential biases. The lack of blinding for participants and intervention facilitators introduces a potential source of performance and expectation bias, although the use of self-reported, validated questionnaires may mitigate this to some extent. Furthermore, our primary reliance on self-reported measures for sensitive outcomes like sexual function and stigma, while necessary and standard, is susceptible to social desirability bias, particularly within a cultural context that discourages open discussion of sexuality. Finally, while we reported overall engagement metrics, the lack of real-time behavioral tracking (e.g., time spent on educational materials, detailed analysis of online interactions) means our fidelity data lacks granularity to fully understand the dose–response relationship and precise mechanisms of engagement.

Future research should aim to address these limitations through larger, multi-center trials with longer follow-up periods (e.g., ≥12 months) to verify the long-term sustainability and generalizability of our intervention model. Building upon this patient-focused foundation, the development and testing of dyadic interventions that actively include partners are a promising and necessary frontier for providing more holistic and impactful support. To enhance methodological rigor, future studies could incorporate objective or behavioral measures where feasible and employ more sophisticated digital tracking to better understand engagement patterns. Finally, exploring the potential of AI-driven personalization (e.g., tailoring content based on individual progress) could further enhance the intervention’s precision and effectiveness.

## 5 Conclusion

In conclusion, this mixed-methods study provides compelling evidence for the efficacy and acceptability of a theory-driven, digital health intervention delivered via social media. By integrating interactive communication principles with patient-centered nursing care, we developed a holistic model that addresses the critical post-operative support gap in sexual health for cervical cancer survivors.

Our findings demonstrate that this accessible digital platform significantly improved key patient-reported outcomes (26). Participants exhibited enhanced sexual function, greater health literacy and empowerment, and reduced stigma. The strong interrelationship among these outcomes underscores the multifaceted nature of recovery, highlighting the value of addressing physiological, psychological, and social dimensions concurrently (27).

This work establishes a practical, scalable, and transferable blueprint for using ubiquitous digital platforms to deliver equitable and sustainable supportive care. It responds to an urgent need in digital public health for innovative strategies that are participatory, culturally sensitive, and responsive to the unmet needs of vulnerable populations. Future efforts should focus on implementing larger, multi-center trials to validate long-term sustainability and on incorporating adaptive technologies, such as AI, for further personalization. Ultimately, this approach paves the way for improving health literacy and quality of life among cancer survivors, contributing to the broader goal of digital health equity.

## Data Availability

The raw data supporting the conclusions of this article will be made available by the authors, without undue reservation.
