# Supplementary material for: A digital health intervention: development and validation of a social media nursing program for sexual dysfunction following cervical cancer radical hysterectomy
Source: Front Public Health. 2025 Dec 4;13:1720263. doi: 10.3389/fpubh.2025.1720263 (PMC12711765; doi:10.3389/fpubh.2025.1720263)
Supplement: Supplementary file 2 [file Supplementary_file_1.docx]

Supplementary File : Study Protocol

Title: A WeChat-Based Digital Nursing Intervention for Sexual Dysfunction in Cervical Cancer Survivors After Radical Hysterectomy: A Randomized Controlled Trial

**1. Background and Objectives**

1.1 Background

Sexual dysfunction is a highly prevalent yet severely under-addressed consequence following radical hysterectomy (RHC) for cervical cancer. Physical sequelae (e.g., vaginal dryness, dyspareunia) and psychological distress (e.g., shame, stigma) significantly impair survivors' quality of life. Cultural taboos and a lack of professional resources create a significant "information support gap" and "communication barriers" for these patients.

**2. Research Question**

Can a theory-driven, social media-based nursing intervention effectively improve sexual function, promote health empowerment, and reduce stigma in cervical cancer survivors experiencing sexual dysfunction after RHC?

**3. Primary Objective**

To evaluate the efficacy of a 5-week WeChat-based intervention on improving sexual function, as measured by the total score of the Female Sexual Function Index (FSFI), in cervical cancer survivors at 3 months post-intervention.

**4. Secondary Objectives**

4.1 To evaluate the intervention's effect on health empowerment, as measured by the Functional Assessment of Cancer Therapy-Cervix (FACT-Cx).

4.2 To evaluate the intervention's effect on reducing perceived stigma, as measured by the Social Impact Scale (SIS).

4.3 To explore the correlations between changes in sexual function, health empowerment, and stigma.

**5. Trial Design**

A prospective, two-arm, randomized controlled trial (RCT) with an allocation ratio of 1:1.

An exploratory sequential mixed-methods design was employed, where this quantitative RCT was informed by a preceding qualitative interview phase.

**6. Participants**

6.1 Inclusion Criteria:(1)Women diagnosed with FIGO stage I-II cervical cancer;

(2)Aged between 18 and 50 years;(3)Had undergone RHC and were >6 months post-surgery;(4)In a stable partnership (defined as married or cohabiting for ≥6 months) with a history of sexual activity;(5)Possessed clear consciousness and normal speech expression ability;(6)Voluntarily participated and provided written informed consent.

6.2 Exclusion Criteria:(1)Mental disorders, cognitive impairments, or other conditions preventing normal communication;(2)Cardiovascular/cerebrovascular diseases or other tumors that could affect sexual life;(3)Pre-existing sexual psychological disorders or sexual dysfunction prior to cancer diagnosis;(4)Patients who, upon confirmation with their attending physician, were not fully informed of their cervical cancer diagnosis.

**7. Interventions**

7.1 Experimental Group: WeChat-Based Intervention.(1)A 5-week program delivered via WeChat, comprising three core components:Interactive Multimedia Education: Weekly push of short videos, infographics, and articles covering anatomical changes, symptom management (vaginal dryness, pain), partner communication, and psychological adaptation.(2)Moderated Peer Support Community: A dedicated, anonymized WeChat group facilitated by a trained research nurse for experience sharing and mutual support.(3)Clinical Specialist-led Counselling: Scheduled live Q&A sessions within the group conducted by gynecologic oncologists and certified sexual health counselors.

7.2 Control Group: Routine Care

Received standard postoperative follow-up care, which focused on monitoring for cancer recurrence and did not include structured assessment, education, or counseling regarding sexual health.

**8. Outcomes**

8.1 Primary Outcome:(1)Measure: Female Sexual Function Index (FSFI) total score.;(2)Tool: 19-item FSFI questionnaire;(3)Time points:Baseline(T0), 1-month post-intervention (T1), 3-months post-intervention (T2).

8.2 Secondary Outcomes

8.2.1 (1)Measure 1: Health Empowerment;(2)Tool: Functional Assessment of Cancer Therapy-Cervix (FACT-Cx);(3)Time points: T0, T1, T2.

8.2.2 (1)Measure 2: Perceived Stigma:(2)Tool: Social Impact Scale (SIS);(3)Timepoints: T0, T1, T2.

**9. Sample Size**

An a priori sample size calculation was performed using G*Power software. Based on the primary outcome (FSFI total score) and anticipating a medium-to-large effect size (f = 0.40) for the group × time interaction in a repeated-measures ANOVA, with α = 0.05 and power (1-β) = 0.80, the analysis indicated a required sample size of 84. Accounting for an estimated 20% attrition rate, the target enrollment was set at 100 participants (50 per group).

**10. Randomization and Blinding**

10.1 Randomization: Eligible participants were randomly assigned to either the intervention or control group using a computer-generated random sequence.

10.2 Blinding: Due to the nature of the behavioral intervention, participants and care providers could not be blinded to the group assignment. However, outcome assessors were blinded as data were collected via self-reported questionnaires.

**11. Statistical Analysis Plan**

11.1 Analysis Sets: The primary analysis will follow the Intent-to-Treat (ITT) principle.

11.2 Descriptive Statistics: Categorical variables will be described with frequencies (n, %) and continuous variables with mean and standard deviation (SD).

11.3 Baseline Comparisons: Baseline characteristics between groups will be compared using Chi-square tests for categorical variables and independent samples t-tests for continuous variables.

11.4 Primary Analysis: The intervention effect on the primary outcome (FSFI total score) will be analyzed using Repeated-Measures Analysis of Variance (RM-ANOVA) to examine the group × time interaction effect. Additionally, Analysis of Covariance (ANCOVA) will be used to compare between-group differences at T1 and T2, controlling for the respective baseline (T0) scores.

11.5 Secondary Analysis: The same RM-ANOVA and ANCOVA approaches will be applied to the secondary outcomes (FACT-Cx and SIS scores).

11.6 Correlation Analysis: Pearson correlation analysis will be conducted to examine the relationships between the FSFI total score, FACT-Cx total score, and SIS total score at the 3-month follow-up (T2).

11.7 Significance Level: A two-sided p-value of < 0.05 will be considered statistically significant.

Software: All analyses will be performed using SPSS 25.0 Statistics

**12. Data Monitoring and Management**

12.1 De-identified data will be stored on a password-protected computer. Access to the final dataset will be limited to the principal investigators.

**13. Ethical Considerations**

13.1 Ethics Approval: This study protocol was approved by the Institutional Review Board of the Affiliated Hospital of Jiangnan University (IRB approval number: WXSY-YXLL-AF/SC-11/02.0).

13.2 Informed Consent: Written informed consent will be obtained from all participants before any study procedures are conducted.

13.3 Protocol Amendments: Any modifications to the protocol which may impact the conduct of the study will require a formal amendment to the protocol and must be approved by the Ethics Committee before implementation.
