# Supplementary material for: A digital health intervention: development and validation of a social media nursing program for sexual dysfunction following cervical cancer radical hysterectomy
Source: Front Public Health. 2025 Dec 4;13:1720263. doi: 10.3389/fpubh.2025.1720263 (PMC12711765; doi:10.3389/fpubh.2025.1720263)
Supplement: Supplementary file 3 [file Table_1.docx]

Supplementary Table 2 Comparison results of general data between the two groups after radical resection of cervical cancer

| **The project** | **Experimental group（n=46）** | **Control group（n=46）** | **Z/*x^2^*** | ***P*** |
| --- | --- | --- | --- | --- |
| **Age (years)** |  |  | -0.41 | 0.68 |
| 18~30 | 12(26.67%) | 11(23.91%) |  |  |
| 31~40 | 16(35.56%) | 22(47.83%) |  |  |
| 41~50 | 17(37.78%) | 13(28.26%) |  |  |
| **Marital status** |  |  | 1.89 | 0.59 |
| unmarried | 8(17.39%) | 8(17.39%) |  |  |
| Married | 15(32.61%) | 16(34.78%) |  |  |
| Get divorced | 17(36.96%) | 12(26.09%) |  |  |
| Widowed spouse | 6(13.04%) | 10(21.74%) |  |  |
| **Place of residence** |  |  |  |  |
| Cities | 26(56.52%) | 25(54.35%) | 0.0 | 1.00 |
| Rural areas | 20(43.48%) | 21(45.65%) |  |  |
| **Degree of education** |  |  | 2.30 | 0.68 |
| Primary school and below | 7(15.22%) | 6(13.04%) |  |  |
| Junior high school | 15(32.61%) | 10(21.74%) |  |  |
| High school or technical secondary school | 11(23.91%) | 14(30.43%) |  |  |
| Junior college | 10(21.74%) | 14(30.43%) |  |  |
| Bachelor degree or above | 3(6.52%) | 2(4.35%) |  |  |
| **Occupations** |  |  | 0.20 | 0.91 |
| individual | 14(30.43%) | 16(34.78%) |  |  |
| On the job | 14(30.43%) | 13(28.26%) |  |  |
| Unemployed or retired | 18(39.13%) | 17(36.96%) |  |  |
| **Monthly household income** |  |  | 5.42 | 0.05 |
| <3000 | 12(26.09%) | 8(17.39%) |  |  |
| 3000~5000 | 8(17.39%) | 12(26.09%) |  |  |
| 5000~8000 | 18(39.13%) | 11(23.91%) |  |  |
| >8000 | 8(17.39%) | 15(32.61%) |  |  |
| **Clinical staging** |  |  | 2.24 | 0.33 |
| Phase I | 28(60.87%) | 27(58.70%) |  |  |
| Phase Ⅱ | 18(39.13%) | 19(41.30%) |  |  |
| **Methods of treatment** |  |  | 1.97 | 0.58 |
| Surgery | 12(26.09%) | 11(23.91%) |  |  |
| Surgery plus chemotherapy | 15(32.61%) | 11(23.91%) |  |  |
| Surgery plus radiotherapy | 12(26.09%) | 12(26.09%) |  |  |
| Surgery plus radiotherapy and chemotherapy | 7(15.22%) | 12(26.09%) |  |  |
| **Postoperative duration (years)** |  |  | 1.91 | 0.38 |
| <1 | 11(23.91%) | 17(36.96%) |  |  |
| 1~3 | 17(36.96%) | 15(32.61%) |  |  |
| >3 | 18(39.13%) | 14(30.43%) |  |  |
| **Resume sexual life time** |  |  | 1.13 | 0.57 |
| ＜Half a year | 16(34.78%) | 21(45.65%) |  |  |
| Six months to one year | 18(39.13%) | 15(32.61%) |  |  |
| >1 year | 12(26.09%) | 10(21.74%) |  |  |
| **Frequency of sexual activity** |  |  | 0.41 | 0.81 |
| More than 4 times per week | 0(0.0) | 0(0.0) |  |  |
| 1 to 4 times per week | 7(15.22%) | 8(17.39%) |  |  |
| Once or twice a month | 20(43.48%) | 17(36.96%) |  |  |
| Less than once a month | 19(41.30%) | 21(45.65%) |  |  |
| **Whether to consult a health care provider about sexual problems** |  |  | 0.70 | 0.40 |
| Yes | 24(52.17%) | 19(41.30%) |  |  |
| No | 22(47.83%) | 27(58.70%) |  |  |
| **Whether to learn about sexual problems through the Internet and other channels** |  |  | 0.71 | 0.40 |
| Yes | 17(36.96%) | 22(47.83%) |  |  |
| No | 29(63.04%) | 24(52.17%) |  |  |
| **Whether to discuss sexual issues with a spouse or partner** |  |  | 0.0 | 1.00 |
| Yes | 27(58.70%) | 26(56.52%) |  |  |
| No | 19(41.30%) | 20(43.48%) |  |  |
