# Supplementary material for: A digital health intervention: development and validation of a social media nursing program for sexual dysfunction following cervical cancer radical hysterectomy
Source: Front Public Health. 2025 Dec 4;13:1720263. doi: 10.3389/fpubh.2025.1720263 (PMC12711765; doi:10.3389/fpubh.2025.1720263)
Supplement: Supplementary file 4 [file Table_2.docx]

Supplementary Table 2 Comparison of FSFI results among patients before intervention

| **The project** | **Before intervention** | | | |
| --- | --- | --- | --- | --- |
|  | **Control group(n=46)** | **Experimental group(n=46)** | ***t*** | ***P*** |
| **Sexual desire** | 3.29±1.20 | 3.12±1.14 | -0.69 | 0.49 |
| **Sexual arousal** | 2.92±0.85 | 2.83±0.95 | -0.45 | 0.65 |
| **Vaginal moisture** | 3.02±1.00 | 3.01±1.20 | -0.03 | 0.98 |
| **Orgasm of sex** | 3.04±1.16 | 2.70±0.92 | -1.56 | 0.12 |
| **Sexual satisfaction** | 2.65±1.24 | 2.33±1.14 | -1.30 | 0.20 |
| **Pain during sexual intercourse** | 2.72±1.04 | 2.46±0.86 | -1.31 | 0.19 |
| **FSFI total score** | 17.64±2.66 | 16.46±3.28 | -1.90 | 0.06 |
