# Supplementary material for: A digital health intervention: development and validation of a social media nursing program for sexual dysfunction following cervical cancer radical hysterectomy
Source: Front Public Health. 2025 Dec 4;13:1720263. doi: 10.3389/fpubh.2025.1720263 (PMC12711765; doi:10.3389/fpubh.2025.1720263)
Supplement: Supplementary file 5 [file Table_3.docx]

Supplementary Table 3 Comparison of FSFI results among patients 1 month after intervention

| **The project** | **One month after the intervention** | | | | |
| --- | --- | --- | --- | --- | --- |
|  | **Control group(n=46)** | **Experimental group(n=46)** | ***t*** | ***P*** | ***Cohen's d*** |
| **Sexual desire** | 2.44±0.76 | 3.16±1.01 | 3.85 | <0.001 | 0.81 |
| **Sexual arousal** | 2.78±0.77 | 2.99±0.89 | 1.20 | 0.23 | 0.25 |
| **Vaginal moisture** | 2.73±0.74 | 3.10±0.84 | 2.26 | 0.03 | 0.47 |
| **Orgasm of sex** | 2.97±1.01 | 2.97±0.70 | 0.00 | 1.00 | 0 |
| **Sexual satisfaction** | 2.68±1.24 | 2.60±0.98 | -0.34 | 0.74 | 0.07 |
| **Pain during sexual intercourse** | 2.39±1.02 | 2.64±0.84 | 1.30 | 0.20 | 0.27 |
| **FSFI total score** | 15.99±2.15 | 17.46±1.96 | 3.43 | <0.001 | 0.71 |
