# Supplementary material for: A digital health intervention: development and validation of a social media nursing program for sexual dysfunction following cervical cancer radical hysterectomy
Source: Front Public Health. 2025 Dec 4;13:1720263. doi: 10.3389/fpubh.2025.1720263 (PMC12711765; doi:10.3389/fpubh.2025.1720263)
Supplement: Supplementary file 6 [file Table_4.docx]

Supplementary Table 4 Comparison of FSFI results among patients 3 months after intervention

| **The project** | **Three months after the intervention** | | | | |
| --- | --- | --- | --- | --- | --- |
|  | **Control group(n=46)** | **Experimental group(n=46)** | ***t*** | ***P*** | ***Cohen's d*** |
| **Sexual desire** | 2.52±1.11 | 3.62±0.76 | 5.56 | <0.001 | 1.16 |
| **Sexual arousal** | 2.77±1.03 | 3.26±0.96 | 2.64 | 0.02 | 0.49 |
| **Vaginal moisture** | 2.64±1.02 | 3.25±0.81 | 2.09 | <0.001 | 0.66 |
| **Orgasm of sex** | 2.75±0.92 | 3.19±0.80 | 2.03 | 0.02 | 0.51 |
| **Sexual satisfaction** | 2.42±1.21 | 3.49±0.97 | 3.33 | <0.001 | 0.98 |
| **Pain during sexual intercourse** | 2.33±1.20 | 2.79±0.77 | 1.86 | 0.03 | 0.46 |
| **FSFI total score** | 15.42±2.67 | 19.60±1.44 | 7.31 | <0.001 | 1.95 |
