# Supplementary material for: A digital health intervention: development and validation of a social media nursing program for sexual dysfunction following cervical cancer radical hysterectomy
Source: Front Public Health. 2025 Dec 4;13:1720263. doi: 10.3389/fpubh.2025.1720263 (PMC12711765; doi:10.3389/fpubh.2025.1720263)
Supplement: Supplementary file 7 [file Table_5.docx]

Supplementary Table 5 Comparison of sexual function recovery between the two groups

|  | **Control group(n=46)** | | **Experimental group(n=46)** | |
| --- | --- | --- | --- | --- |
|  | **One month after the intervention** | **Three months after the intervention** | **One month after the intervention** | **Three months after the intervention** |
| **1** | -0.85±1.42(-25.84%) | -0.77±1.63(-23.4%) | 0.04±1.52(1.28%) | 0.5±1.37(16.03%) |
| **2** | -0.14±1.15(-4.79%) | -0.15±1.34(-5.14%) | 0.16±1.3(5.65%) | 0.43±1.35(15.19%) |
| **3** | -0.29±1.24(9.6%) | -0.38±1.43(-12.58%) | 0.09±1.46(2.99%) | 0.24±1.45(7.97%) |
| **4** | -0.07±1.54(-2.3%) | -0.29±1.48(-9.54%) | 0.27±1.16(10.0%) | 0.49±1.22(18.15%) |
| **5** | 0.03±1.75(1.13%) | -0.23±1.73(-8.68%) | 0.27±1.5(11.59%) | 1.16±1.5(49.79%) |
| **6** | -0.33±1.46(-12.13%) | -0.39±1.59(-14.34%) | 0.18±1.2(7.32%) | 0.33±1.15(13.41%) |
| **7** | -1.65±3.42(-9.35%) | -2.22±3.77(-12.59%) | 1.0±3.82(6.08%) | 3.14±3.58(19.08%) |
| Note: 1. Sexual desire; 2. Sexual arousal; 3. Vaginal wetness; 4. Orgasm; 5. Sexual satisfaction; 6. Pain during sexual intercourse; 7. FSFI total score. | | | | |
