# Supplementary material for: A digital health intervention: development and validation of a social media nursing program for sexual dysfunction following cervical cancer radical hysterectomy
Source: Front Public Health. 2025 Dec 4;13:1720263. doi: 10.3389/fpubh.2025.1720263 (PMC12711765; doi:10.3389/fpubh.2025.1720263)
Supplement: Supplementary file 8 [file Table_6.docx]

Supplementary Table 6 FACT-Cx and SIS questionnaire scores before intervention

|  | **Control group(n=46)** | **Experimental group(n=46)** | **Z/t** | ***P*** |
| --- | --- | --- | --- | --- |
| **Self-management** | 21.15±4.16 | 20.93±5.16 | -0.22 | 0.82 |
| **Life attitude** | 24.87±5.85 | 25.72±6.41 | 0.66 | 0.51 |
| **Obtaining support** | 19.52±4.14 | 19.76±4.66 | 0.26 | 0.80 |
| **Acceptance and inclusion** | 10.26±2.60 | 10.61±3.01 | 0.59 | 0.55 |
| **Health empowerment** | 75.80±8.56 | 77.02±9.10 | 0.66 | 0.51 |
| **Stigma score** | 67.37±15.8 | 61.37±19.46 | -1.53 | 0.13 |
