# Supplementary material for: A digital health intervention: development and validation of a social media nursing program for sexual dysfunction following cervical cancer radical hysterectomy
Source: Front Public Health. 2025 Dec 4;13:1720263. doi: 10.3389/fpubh.2025.1720263 (PMC12711765; doi:10.3389/fpubh.2025.1720263)
Supplement: Supplementary file 9 [file Table_7.docx]

Supplementary Table 7 Scores on the FACT-Cx and SIS questionnaires one month after the intervention

|  | **Control group(n=46)** | **Experimental group(n=46)** | **Z/t** | ***P*** | ***Cohen's d*** |
| --- | --- | --- | --- | --- | --- |
| **Self-management** | 16.20±2.23 | 24.09±4.34 | 10.98 | <0.001 | 1.89 |
| **Life attitude** | 16.61±3.70 | 25.65±6.13 | 8.51 | <0.001 | 1.79 |
| **Obtaining support** | 15.09±4.43 | 20.76±3.61 | 6.73 | <0.001 | 1.40 |
| **Acceptance and inclusion** | 10.54±2.54 | 12.80±1.65 | 5.06 | <0.001 | 1.06 |
| **Health empowerment** | 58.43±7.11 | 83.30±5.62 | 18.73 | <0.001 | 1.88 |
| **Stigma score** | 62.33±14.76 | 55.80±11.06 | -1.68 | 0.1 | 0.50 |
