# Supplementary material for: A digital health intervention: development and validation of a social media nursing program for sexual dysfunction following cervical cancer radical hysterectomy
Source: Front Public Health. 2025 Dec 4;13:1720263. doi: 10.3389/fpubh.2025.1720263 (PMC12711765; doi:10.3389/fpubh.2025.1720263)
Supplement: Supplementary file 10 [file Table_8.docx]

Supplementary Table 8 Scores on the FACT-Cx and SIS questionnaires three months after the intervention

|  | **Control group(n=46)** | **Experimental group(n=46)** | **Z/t** | ***P*** | ***Cohen's d*** |
| --- | --- | --- | --- | --- | --- |
| **Self-management** | 15.70±1.82 | 26.93±5.65 | 12.85 | <0.001 | 2.68 |
| **Life attitude** | 14.48±2.39 | 26.26±6.55 | 11.46 | <0.001 | 2.39 |
| **Obtaining support** | 12.41±3.22 | 21.57±3.80 | 12.45 | <0.001 | 2.60 |
| **Acceptance and inclusion** | 10.39±2.53 | 13.02±1.60 | 5.95 | <0.001 | 1.24 |
| **Health empowerment** | 52.98±5.86 | 87.78±5.60 | 29.13 | <0.001 | 2.07 |
| **Stigma score** | 62.48±15.09 | 44.46±10.01 | -4.05 | <0.001 | 1.41 |
