# Supplementary material for: A digital health intervention: development and validation of a social media nursing program for sexual dysfunction following cervical cancer radical hysterectomy
Source: Front Public Health. 2025 Dec 4;13:1720263. doi: 10.3389/fpubh.2025.1720263 (PMC12711765; doi:10.3389/fpubh.2025.1720263)
Supplement: Supplementary file 11 [file Table_9.docx]

Supplementary Table 9 Comparison table of health empowerment and stigma of patients after intervention

|  | **Control group(n=46)** | | **Experimental group(n=46)** | |
| --- | --- | --- | --- | --- |
|  | **One month after the intervention** | **Three months after the intervention** | **One month after the intervention** | **Three months after the intervention** |
| **1** | -4.95±4.72(-23.4%) | -5.45±4.54(-25.77%) | 3.16±6.74(15.1%) | 6.0±7.65(28.67%) |
| **2** | -8.2±6.92(-32.97%) | -10.39±6.32(-41.78%) | -0.07±8.87(-0.27%) | 0.54±9.16(2.1%) |
| **3** | -4.43±6.06(-22.82%) | -7.11±5.24(-36.42%) | 1.0±5.89(5.06%) | 1.81±6.01(9.16%) |
| **4** | 0.28±3.63(2.73%) | 0.13±3.63(1.27%) | 2.19±3.43(20.63%) | 2.41±3.41(22.71%) |
| **5** | -17.3±11.06-22.82%) | -22.82±10.37(-30.11%) | 6.28±10.7(8.15%) | 10.76±10.69(13.97%) |
| **6** | -5.61±22.26(-8.33%) | -5.98±22.37(-8.88%) | -4.98±23.29(-8.07%) | -12.46±23.23(-20.19%) |
| Notes: 1. Self-management score; 2. Life attitude score; 3. Score of obtaining support; 4. Acceptance and inclusion score; 5. Health empowerment score;6.Stigma score. | | | | |
