# Supplementary material for: A digital health intervention: development and validation of a social media nursing program for sexual dysfunction following cervical cancer radical hysterectomy
Source: Front Public Health. 2025 Dec 4;13:1720263. doi: 10.3389/fpubh.2025.1720263 (PMC12711765; doi:10.3389/fpubh.2025.1720263)
Supplement: Supplementary file 12 [file Table_10.docx]

Supplementary Table 10. ANCOVA Results for Primary and Secondary Outcomes at 1-Month and 3-Month Follow-ups.

| **Outcome Measure** | **Time Point** | **Group (Adjusted Mean ± SE)** | | **F-value** | **p-value** | **Effect Size (Partial η²)** |
| --- | --- | --- | --- | --- | --- | --- |
|  |  | Intervention | Control |  |  |  |
| **FSFI Total Score** | 1 Month | 17.46 ± 0.29 | 15.99 ± 0.29 | 14.80 | <0.001 | 0.143 |
|  | 3 Months | 19.60 ± 0.26 | 15.42 ± 0.26 | 53.44 | <0.001 | 0.375 |
| **FACT-Cx** | 1 Month | 83.30 ± 0.82 | 58.43 ± 0.82 | 451.27 | <0.001 | 0.835 |
|  | 3 Months | 87.78 ± 0.82 | 52.98 ± 0.82 | 1023.16 | <0.001 | 0.920 |
| **SIS Total Score** | 1 Month | 55.80 ± 1.84 | 62.33 ± 1.84 | 6.83 | <0.001 | 0.071 |
|  | 3 Months | 44.46 ± 1.84 | 62.48 ± 1.84 | 50.39 | <0.001 | 0.362 |
