# Supplementary material for: A digital health intervention: development and validation of a social media nursing program for sexual dysfunction following cervical cancer radical hysterectomy
Source: Front Public Health. 2025 Dec 4;13:1720263. doi: 10.3389/fpubh.2025.1720263 (PMC12711765; doi:10.3389/fpubh.2025.1720263)
Supplement: Supplementary file 13 [file Table_11.docx]

Supplementary Table 11 theory-intervention matrix

| **Theoretical Construct (Nutbeam)** | **Intervention Component** | **Operationalization via WeChat Features** | **Intended Outcome** |
| --- | --- | --- | --- |
| Functional Health Literacy (Basic knowledge &understanding) | Interactive Multimedia Education | Weekly push of short videos,infographics,and articles via WeChat Official Account or private chats. | Improved knowledge of post-op anatomy,symptoms,and management techniques. |
| Interactive Health Literacy (Social &communication skills) | Moderated Peer Support Community | A dedicated,anonymized WeChat group facilitated by a research nurse. | Enhanced communication skills,reduced isolation,peer-to-peer learning. |
| Critical Health Literacy (Critical analysis,self-efficacy,empowerment) | Clinical Specialist-led Counselling &Overall Program | Scheduled live Q&A sessions in the group;reflective activities encouraged by the moderator. | Increased confidence in managing health,improved self-advocacy,reduced stigma. |
